# Cavity-enhanced photoacoustic dual-comb spectroscopy

**DOI:** 10.1038/s41377-023-01353-6

**Published:** 2024-01-05

**Authors:** Zhen Wang, Qinxue Nie, Haojia Sun, Qiang Wang, Simone Borri, Paolo De Natale, Wei Ren

**Affiliations:** 1 Department of Mechanical and Automation Engineering, The Chinese University of Hong Kong, New Territories, Hong Kong SAR, China; 2 State Key Laboratory of Applied Optics, Changchun Institute of Optics, Fine Mechanics and Physics, Chinese Academy of Sciences, 130033 Changchun, China; 3 CNR-INO—Istituto Nazionale di Ottica, and LENS—European Laboratory for Nonlinear Spectroscopy, 50019 Sesto Fiorentino, Italy

**Keywords:** Optical sensors, Infrared spectroscopy

## Abstract

Photoacoustic dual-comb spectroscopy (DCS), converting spectral information in the optical frequency domain to the audio frequency domain via multi-heterodyne beating, enables background-free spectral measurements with high resolution and broad bandwidth. However, the detection sensitivity remains limited due to the low power of individual comb lines and the lack of broadband acoustic resonators. Here, we develop cavity-enhanced photoacoustic DCS, which overcomes these limitations by using a high-finesse optical cavity for the power amplification of dual-frequency combs and a broadband acoustic resonator with a flat-top frequency response. We demonstrate high-resolution spectroscopic measurements of trace amounts of C_2_H_2_, NH_3_ and CO in the entire telecommunications C-band. The method shows a minimum detection limit of 0.6 ppb C_2_H_2_ at the measurement time of 100 s, corresponding to the noise equivalent absorption coefficient of 7 × 10^−10 ^cm^−1^. The proposed cavity-enhanced photoacoustic DCS may open new avenues for ultrasensitive, high-resolution, and multi-species gas detection with widespread applications.

## Abstract (web summary)

We demonstrate cavity-enhanced photoacoustic dual-comb spectroscopy with a flute-type acoustic resonator and an optical cavity for spectral measurements with ultra-high sensitivity, high resolution, and broad bandwidth.

## Graphical abstract

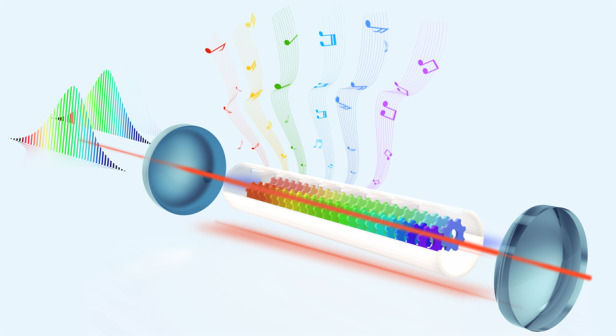

## Introduction

Since its first demonstrations two decades ago^1–4^, dual-comb spectroscopy (DCS) has evolved into a powerful tool in many fields such as spectroscopy and microscopy^5–9^, precision metrology^10,11^, spectral lidar^12,13^, environmental monitoring^14,15^, and advanced hyperspectral holography and imaging^16,17^. Particularly, DCS plays an important role in modern high-precision and broadband molecular spectroscopy, which performs Fourier transform spectroscopy rapidly without using any moving parts. In this setup, one frequency comb passes through a gas sample to be analyzed and beats on a photodetector with a second phase-locked comb with a slightly different repetition rate. The multiheterodyne beats between pairs of comb lines generate an interferometric signal, which is accessible by radio-frequency (RF) electronics and Fourier-transformed to reveal the sample’s spectrum. DCS can fully capitalize on the frequency resolution and accuracy, broad bandwidth, and high repetition rate of different types of comb sources for high-speed, ultrahigh-resolution and broadband spectroscopy^6,7,18–21^.

Conventional DCS^22^ is implemented by measuring the transmitted comb light intensity precisely using a fast photodetector. The absorption spectrum needs to be extracted from the large background signal, which is not a trivial task especially for weak absorbance. Compared to direct absorption measurements, DCS can also be performed by taking advantage of other spectroscopic techniques such as photoacoustic and photothermal detection^23–26^. These indirect absorption measurements enable the background-free detection of molecular spectra, where only the comb lines absorbed by the gas medium can generate the photoacoustic/thermal multi-heterodyne beatnotes.

For instance, photoacoustic spectroscopy (PAS), which has been widely used in gas sensing^27^ and imaging^28^, normally uses a microphone to detect acoustic waves, which are generated by the non-radiative collisional relaxation of the excited molecules after absorbing the modulated light. Sadiek et al. reported the first PAS using a frequency comb and implemented a Fourier transform spectrometer (FTS) to modulate the intensity of the frequency comb^29^. To eliminate the mechanical parts in FTS, photoacoustic DCS has been recently demonstrated for measuring gaseous acetylene (C_2_H_2_)^23^ and polymer films^24^. In these proof-of-concept experiments, the detection sensitivity still needs to be improved, i.e., a minimum detection limit (MDL) of 10 ppm was achieved for C_2_H_2_ detection at a recording time of 1000 s^23^. By replacing the microphone with a quartz tuning fork (QTF) that has a high Q-factor, the QTF-based photoacoustic DCS was developed to improve the MDL to 8.3 ppb C_2_H_2_^30^. Nevertheless, the generated RF comb lines have to lie within the extremely narrow resonance bandwidth (several Hz) of the QTF, significantly limiting the detection bandwidth. Therefore, the capability of achieving the simultaneous high sensitivity and broad bandwidth for photoacoustic DCS has been hindered by lacking high-power comb light for exciting the photoacoustic effect and broadband acoustic resonators for amplifying all the generated acoustic waves effectively.

In this study, we introduce cavity-enhanced photoacoustic DCS for ultrasensitive, broadband, and high-resolution spectroscopic detection, by combining two cutting-edge technologies to overcome the aforementioned shortcomings. First, employing a flute-type acoustic resonator, we realize sensitive photoacoustic detection with a 3-dB bandwidth of more than 5 kHz in the audio frequency range of 2.7–8.0 kHz. Second, injecting the two trains of frequency combs simultaneously into a high-finesse optical cavity enables the intracavity power build-up by several orders of magnitude. This leverages the broad detection bandwidth of the novel acoustic resonator and the remarkable comb power enhancement afforded by the optical cavity. In the experiment, we demonstrate the high-resolution cavity-enhanced photoacoustic DCS of multiple gas-phase species including C_2_H_2_, NH_3_ and CO in the entire telecommunications C-band with an ultra-high sensitivity among the state-of-the-art frequency comb spectroscopy.

## Results

### Concept

**Fig. 1:**
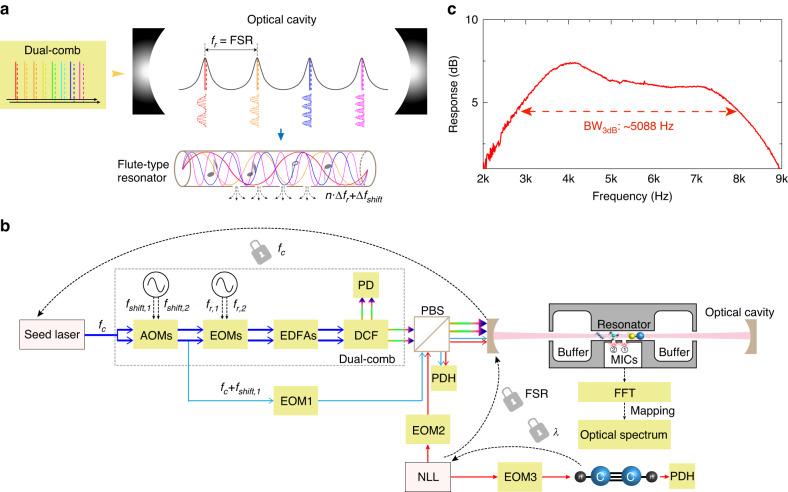
Cavity-enhanced photoacoustic dual-comb spectroscopy. **a** Concept of the cavity-enhanced photoacoustic DCS approach. The dual-frequency combs are coupled into an optical cavity for power enhancement when all the comb lines exhibit a perfect match with the cavity modes. After absorption by the target gas molecules, the multi-heterodyne of the intracavity dual-combs generates multiple acoustic waves with the frequencies determined by the difference of repetition rates (△*f*_r_) and central frequency shifts (△*f*_shift_). A flute-type acoustic resonator with a broadband frequency response is designed to amplify the generated photoacoustic waves. **b** Schematic of the experimental setup. The electro-optic dual-comb source employs a CW seed laser at optical frequency *f*_c_, which is divided into two branches and connected in parallel with pairs of acousto-optic modulators (AOMs) to control the central frequency shifts (*f*_shift,1_ and *f*_shift,2_) and electro-optic modulators (EOMs) to control the repetition rates (*f*_r,1_ and *f*_r,2_). The generated optical pulses are then amplified by two Erbium-doped fiber amplifiers (EDFAs) and counter-launched into a nonlinear dispersion compensated fiber (DCF). A photodetector (PD) is used with the dual-comb source to monitor the multiheterodyne reference spectrum. Three Pound-Drever-Hall (PDH) locking loops are used to match the comb lines with the cavity modes: the AOM-shifted seed laser is phase-modulated by EOM1 (19 MHz) to stabilize the carrier frequency (*f*_*c*_) with respect to the Fabry–Pérot cavity; a narrow-linewidth laser (NLL) is phase-modulated by EOM2 (13 MHz) to lock with the optical cavity and by EOM3 (99 MHz) to lock with an absorption line of C_2_H_2_ at 10 Torr, respectively. The two CW lasers are arranged in orthogonal polarization with the dual-comb light to avoid optical crosstalk, and combined via a polarization beam splitter (PBS) before entering the optical cavity. The broadband acoustic resonator is situated inside the optical cavity for acoustic wave amplification and two microphones (MICs) are used to detect the acoustic waves. **c** Characterized frequency response of the broadband acoustic resonator. The resonator shows a 3-dB bandwidth (BW_3dB_) of 5088 Hz

The working principle of cavity-enhanced photoacoustic DCS is illustrated in Fig. 1a. Different from the dual-comb absorption measurement in an optical cavity^31–34^, cavity-enhanced photoacoustic DCS requires the two combs to be coupled into a cavity simultaneously to enable the generation of intracavity dual-comb multiheterodyne beatnotes. Provided both combs enter the cavity effectively for power enhancement, the multi-heterodyne beating between each comb-line pair causes an amplitude modulation of the cavity-enhanced comb line. After absorption by the target molecules, this leads to the excitation of hundreds or thousands of acoustic waves with evenly spaced frequencies, determined by the repetition rate difference (△*f*_r_) and central frequency shifts (△*f*_shift_) between the two phase-coherent combs^23,26^. Ideally, a broadband and open-ended acoustic resonator, which is situated inside the optical cavity, further amplifies all the acoustic waves for more sensitive detection.

### Setup

The schematic of the experimental setup is shown in Fig. 1b. Here an electro-optic dual-comb source is used for demonstration purposes considering its flexible tuning of repetition rate (*f*_r_) and optical carrier frequency (*f*_c_)^35^. More details of the dual-comb source are provided in Methods. The two trains of frequency combs generated from the same continuous wave (CW) seed laser share the same (not constant) carrier frequency. For one train of the frequency combs with the repetition rate *f*_r,1_, locking the seed laser (*f*_c_) to the Fabry–Pérot cavity enclosed in a gas cell enables the overlap between the central comb line and one cavity mode, which can be conducted using the Pound-Drever-Hall (PDH) technique^36^. The other comb lines are coupled into the cavity by tuning *f*_r,1_ so that it perfectly matches the free spectral range (FSR, ~833 MHz) of the optical cavity. To obtain a temporally invariant FSR, a stable narrow-linewidth CW laser (1531.58 nm) shown in Fig. 1b is employed as an optical intermedium to stabilize the cavity length by locking the cavity mode to an absorption line of C_2_H_2_ (Supplementary Note 1). As a result, all the comb lines with repetition rate *f*_r,1_ are arranged in perfect resonance with the cavity modes, whereas the counterpart of the dual-comb source with a slight difference in the repetition rate (*f*_r,2_ = *f*_r,1_ + △*f*_r_, where △*f*_r_ = 30 Hz) can enter the cavity automatically. We estimate a negligible difference (< 0.7%) in the relative intensity attenuation for the two comb lines coupled into the same cavity mode (Methods). Additionally, we eliminated the possible interference between the three locking loops by using an orthogonal polarization arrangement for the comb light and CW lasers and carefully selecting modulation frequencies for the three EOMs used for PDH locking purposes (Supplementary Note 1).

A broadband acoustic detector serves as a key element in photoacoustic DCS. Inspired by the flute instrument, we designed a broadband acoustic resonator to amplify many acoustic waves with distinct frequencies. As shown in Fig. 1b, it includes a longitudinal acoustic resonator (length: 35 mm; inner diameter: 2 mm) in the center, connected with two buffering volumes (length: 17.5 mm; inner diameter: 12 mm). Two end-caps are used to cover the buffering volumes, and a central through hole (diameter: 2 mm) is made in each cap for optical access. For acoustic wave detection, two microphones are installed in the central acoustic resonator and the superimposed electrical signals are added by a low-noise summing circuit. Finally, the microphone output is digitized and Fourier-transformed to obtain the photoacoustic spectrum. It should be noted that all the signal generators and the data-acquisition card are synchronized to a rubidium clock to maintain long-term locking and measurement stability.

The frequency response of the broadband acoustic resonator was characterized by measuring the photoacoustic signal of 10 ppm C_2_H_2_/N_2_ using a 1531.58-nm CW laser at varied intensity modulation frequencies (Supplementary Note 2). As shown in Fig. 1c, our acoustic resonator shows an excellent flat-top frequency response with a bandwidth (3 dB) of 5088 Hz in the frequency range of 2.9-8.0 kHz. This is tens of times broader than the traditional longitudinal acoustic resonator^37^, and over three orders of magnitude larger than the QTF^30,38^. As discussed later, such a broadband response benefits from the merging of higher-order acoustic modes inside the acoustic resonator.

**Fig. 2:**
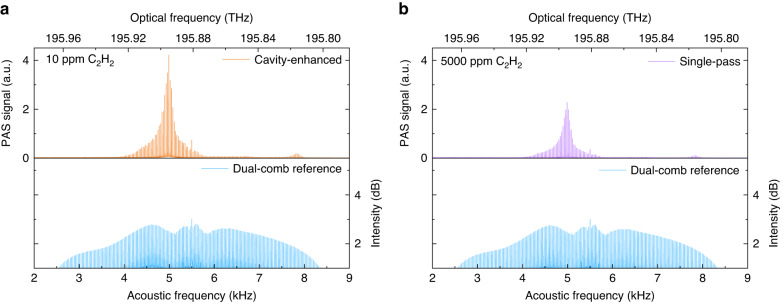
Comparison of photoacoustic dual-comb spectra obtained with and without an optical cavity. **a** Representative cavity-enhanced photoacoustic DCS signal of 10 ppm C_2_H_2_ (top panel) and the corresponding incident intensity of the dual-comb (bottom panel). **b** Representative single-pass photoacoustic DCS signal of 5000 ppm C_2_H_2_ (top panel) and the corresponding incident intensity of the dual-comb (bottom panel). The measurements are averaged over 60 s. The acoustic frequency scale (kHz) is converted to the optical domain based on the frequency compression factor *f*_r_/Δ*f*_r_ and the carrier frequency *f*_c_. A strong absorption line of C_2_H_2_ at 195.895 THz and a weak line at 195.818 THz are observed in this frequency range

By filling 10 ppm C_2_H_2_ in the gas cell at the atmospheric pressure (760 Torr), the representative cavity-enhanced photoacoustic DCS signal is illustrated in the top panel of Fig. 2a. The strong spectral profile at 5 kHz in the acoustic frequency domain corresponds to the *P*(9) line of C_2_H_2_ at 195.895 THz in the optical domain. In contrast, we also conducted the single-pass measurement by using the same dual-comb source and acoustic resonator, but removing the optical resonator from the setup. Note that a much higher concentration (5000 ppm) of C_2_H_2_ was required to achieve a similar signal level in Fig. 2b. The dual-comb reference spectra plotted in the two bottom panels were recorded when measuring the single-pass signal and the cavity-enhanced signal, respectively; they show similar profiles in these two measurements. By taking into account the difference in the photoacoustic signal amplitude and gas concentration, the use of such a high-finesse cavity significantly enhances the dual-comb signal by a factor of 924. We estimate an average intracavity comb power of 130 mW for each pair of comb lines (Methods).

### Broadband multi-species measurement

**Fig. 3:**
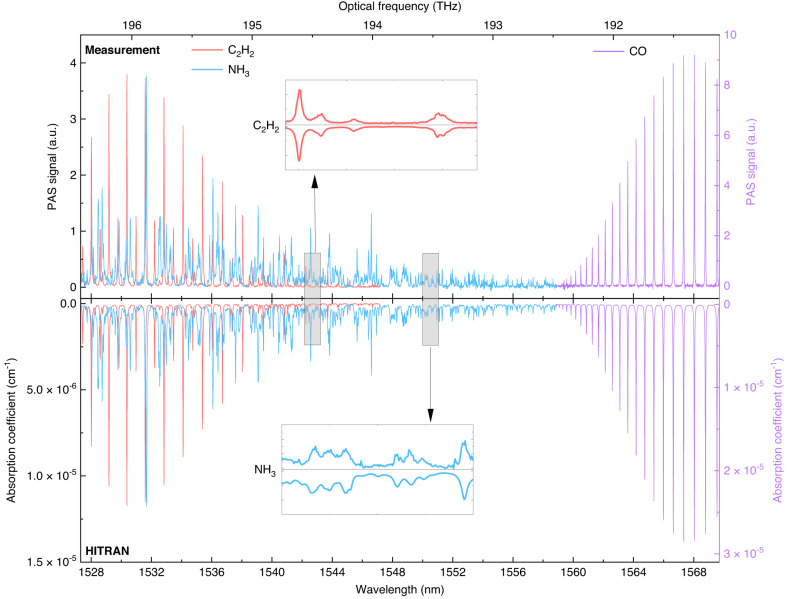
Broadband molecular spectra of multiple species. Cavity-enhanced photoacoustic DCS of 10 ppm C_2_H_2_, 50 ppm NH_3_ and 1% CO is measured over the telecommunications C-band at the atmospheric pressure (760 Torr). The spectral simulation based on the HITRAN database is also plotted for comparison. Insets: weak signatures of C_2_H_2_ and NH_3_ resolved by the cavity-enhanced photoacoustic dual-comb system

Over the telecommunications C-band (1527.3–1569.7 nm), we conducted cavity-enhanced photoacoustic DCS measurements of 10 ppm C_2_H_2_, 50 ppm NH_3_ and 1% CO at the pressure of 760 Torr, respectively. The measurement was recorded with a data sampling rate of 500 kS/s and FFT resolution of 1 Hz. The photoacoustic spectrum was averaged over 60 s to improve the signal-to-noise ratio (SNR), followed by the amplitude normalization by the non-uniform comb-power envelope, the variation of cavity finesse over the wide spectral range, and the frequency response of the acoustic resonator (Supplementary Note 3). Figure 3 shows the entire wide spectra by stitching 41 photoacoustic spectra of C_2_H_2_, 66 photoacoustic spectra of NH_3_ and 21 photoacoustic spectra of CO mixtures measured with an SNR (Methods) of 5952, 8621 and 347, respectively. All the measurements are in good agreement with the simulated absorption spectra using the HITRAN database^39^. In particular, the inset graph demonstrates several very weak lines of C_2_H_2_ with absorption coefficients around 1 × 10^−6 ^cm^−1^. The polyatomic molecule NH_3_ features a complex infrared spectrum with many blended lines, which are well resolved by our spectrometer as shown in the inset graph. Note that the overtone spectrum of CO in this wavelength range has a very small line-strength (mostly below 10^−23 ^cm^−1^/(molecules cm^−2^)), which is 2–3 orders of magnitude smaller than C_2_H_2_ and NH_3_.

### Linear response and detection limit

**Fig. 4:**
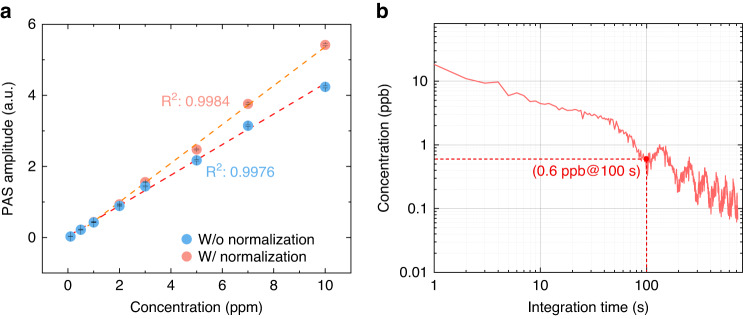
Experimental results for linear response and detection limit. **a** Variation of the photoacoustic amplitude with C_2_H_2_ concentration. The system shows a good linear response with an *R*^2^ value of 0.9976, after normalization, the linearity can be better with a higher *R*^2^ value of 0.9984. The vertical error bars (1-σ standard deviation) are calculated from the raw data, taken in a time interval of 60 s. **b** Allan-Werle deviation analysis of C_2_H_2_ detection. The measurement was conducted by recording the system response of pure N_2_ for one hour with all locking loops turned on

The strongest absorption line of each species was selected to investigate the gas sensing performance. Here we studied the *P*(9) line of C_2_H_2_ at 195.895 THz with a line-strength of 1.211 × 10^−20 ^cm^−1^/(molecules cm^−2^). Note that the cavity finesse may degrade due to the stronger absorption at a much higher gas concentration, thus affecting the linear response of cavity-enhanced gas sensors^40,41^. With this factor taken into account, Fig. 4a plots the amplitude of the photoacoustic signal as a function of gas concentration for C_2_H_2_/N_2_ mixtures, showing a good linear response (*R*^2^ > 0.99). The vertical error bar (1-σ standard deviation) is calculated from the variation of the peak amplitude acquired over a time period of 60 s. The Allan-Werle deviation analysis is conducted to evaluate the long-term stability and detection limit by measuring zero gas (here N_2_)^40–42^ for one hour. The photoacoustic signal and the noise are evaluated at the dual-comb power of 30 mW and the same acoustic frequency of 5290 Hz which corresponds to the absorption peak of C_2_H_2_ at 195.895 THz (Methods). As illustrated in Fig. 4b, our sensor demonstrates a minimum detection limit (MDL) of 0.6 ppb for C_2_H_2_ at the averaging time of 100 s, corresponding to the noise equivalent absorption (NEA) coefficient of 7 × 10^−10 ^cm^−1^. We also studied the blended lines (^*p*^*P*(5,3)*s*, ^*p*^*P*(5,3)*a*) of NH_3_ at 195.731 THz with a line-strength of 1.35 × 10^−21 ^cm^−1^/(molecules cm^−2^) and the *R*(7) line of CO at 191.190 THz with a line-strength of 2.22 × 10^−23 ^cm^−1^/(molecules cm^−2^). The results are provided in Supplementary Note 4.

## Discussion

**Fig. 5:**
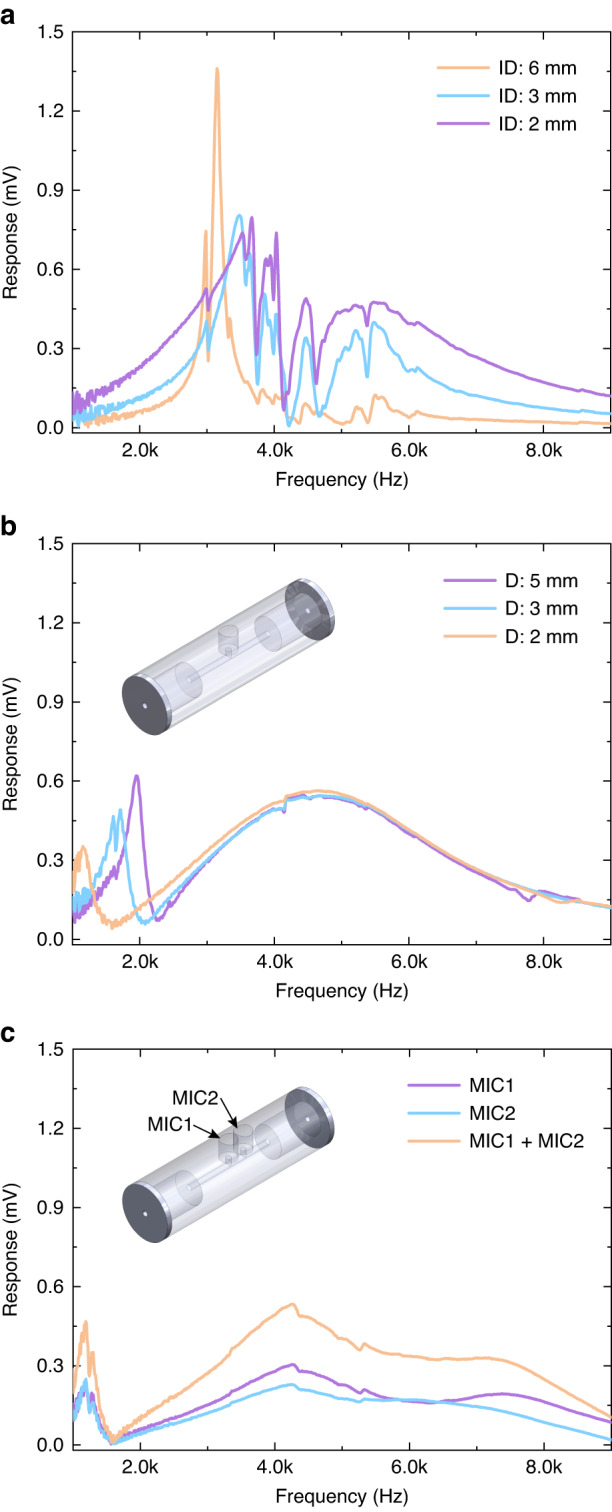
Characterization of different types of acoustic resonators. **a** Frequency responses of the traditional acoustic resonators with different inner diameters (IDs). **b** Frequency responses of the traditional acoustic resonators with end caps. The ID of the central resonator is fixed at 2 mm, while the diameter (D) of the through holes in the end caps is varied between 2 and 5 mm. Inset: 3D drawing of the acoustic resonator. **c** Frequency responses of the flute-type acoustic resonator with different configurations of microphones. Inset: 3D drawing of the acoustic resonator

The flute-type acoustic resonator plays a significant role in cavity-enhanced photoacoustic DCS for broadband acoustic detection. Here we briefly discuss the parameters affecting its frequency response; the detailed structure of the acoustic resonator is provided in Supplementary Note 2. Figure 5a illustrates the different frequency responses of an open-ended longitudinal resonator with varied inner diameters. The resonator with an inner diameter of 2 mm excites more high-frequency acoustic modes. Two end caps are then added to cover the buffering volumes with a diameter of 12 mm; a central hole is made for optical access. As shown in Fig. 5b, it leads to a smooth and broadband frequency response. The configuration with a smaller hole (2 mm diameter) in the cap contributes to a broader frequency response (4108 Hz in bandwidth). Finally, compared to the single microphone installed at the central position shown in Fig. 5a and b, we drilled another hole nearby (6.5 mm away) for the installation of the second microphone. The generated electrical signals by the two microphones are summed by a low-noise circuit with the results plotted in Fig. 5c, showing a flat-top response with an increased bandwidth of 5088 Hz. The traditional photoacoustic resonator is characterized by a Q-factor of 38 and bandwidth of 150 Hz. Such a narrow bandwidth makes it unsuitable for amplifying multiple acoustic waves. In comparison, our resonator has a bandwidth of 5088 Hz, which is about 34 times larger. By targeting a broader flat-top frequency response with a larger signal amplitude, it is possible to further optimize the geometry of the acoustic resonator using finite element analysis^27,43^. It is also interesting to explore acoustic resonators with bandwidths located at higher frequencies to reduce the 1/*f* noise.

Our platform proves the feasibility of significantly enhancing the comb power by coupling both frequency combs into a high-finesse optical cavity. The successful implementation relies on the one-to-one matching between the comb line and the cavity mode, enabled by the precise control of the comb’s two degrees of freedom (*f*_r_ and *f*_c_) and the cavity length. The average optical power of each comb pair inside the cavity amounts to 130 mW in this work, which can be further enhanced by using a higher-finesse cavity or a high Q-factor microring resonator^44^. As the simultaneous injection of dual combs into the cavity is needed for intracavity multi-heterodyne beating, the sharper resonance of the higher-finesse cavity may cause certain intensity attenuation for the comb line that is slightly off the cavity resonance. By applying an optical cavity with ten times larger finesse (40780) but reducing the cavity length from 18 cm to 6 cm, the FSR triples to 2.5 GHz and the cavity mode width reduces to 61 kHz. This will only cause a slight attenuation of (< 7%) of the comb intensity for the comb line index beyond 200 (Methods).

Additionally, trace gas detection favors spectral measurements in the mid-infrared region. As mid-infrared dual-combs can be generated using the difference frequency generation (DFG) of a near-infrared electro-optic comb^45^, we can readily extend the method proposed in the current study to mid-infrared gas sensing applications. Although other types of mid-infrared frequency combs have been recently invented^6,7,19,46–50^, one may devote to achieving the spectral overlap between the comb lines and cavity modes considering the different mechanisms of controlling the comb parameters. For instance, the mid-infrared combs generated by the DFG process^6^ need extra mechanisms to match with the optical cavity. Another type of mid-infrared combs created in synchronously pumped optical parametric oscillators (OPOs)^7^ requires additional electronics to stabilize the repetition rate difference and carrier-envelope offset. For quantum-cascade-laser (QCL) frequency combs^19^, injection locking and phase locking loops are needed to fully stabilize the comb spacing and frequency offset^51^. Note that QCL combs normally have a mode spacing of ~10 GHz which are more suitable for spectral measurements of large molecules. Hence, although demonstrated using electro-optic combs in this work, we expect our cavity-enhanced PAS approach can be realized using other fully stabilized comb sources to meet different demands.

In conclusion, we present a novel spectroscopic technique, cavity-enhanced photoacoustic DCS, for ultrasensitive, broadband, and high-resolution molecular spectroscopy and trace gas detection. The acoustic resonator shows a bandwidth beyond 5 kHz which is tens of times broader than the traditional one and ~1000 times broader than the tuning fork. With a coupling efficiency beyond 90%, the high-finesse cavity enhances the optical power of hundreds of comb pairs simultaneously by nearly three orders of magnitude. Benefiting from the broadband acoustic resonator and high-finesse optical cavity, our method enables the comb-line-resolved DCS measurement of trace amounts of C_2_H_2_, NH_3_ and CO in the entire telecommunications C-band. Compared to the recent results of photoacoustic and photothermal DCS of C_2_H_2_^22,26^, we have improved the detection sensitivity remarkably from ppm to sub-ppb level. These unique features of our cavity-enhanced photoacoustic DCS may enable a highly powerful analytical tool for broadband, high-precision and high-sensitivity spectroscopic measurements and gas sensing applications.

## Materials and methods

### Dual-comb source

The electro-optic comb is seeded by a CW external cavity diode laser emitting at optical frequency *f*_c_. The seed laser is divided into two branches and each is connected in parallel to an AOM shifting the optical frequency by 25 MHz and 25.0055 MHz, respectively. This leads to a center frequency of 5.5 kHz for the multiheterodyne beatnotes. The frequency comb is generated by intensity modulation using an EOM, which is driven by 50-ps pulses at the repetition rates of 832.95250 MHz and 832.95253 MHz, respectively. Thus we obtain a frequency spacing of 30 Hz for the multiheterodyne beats. After passing through EDFAs, the two frequency combs are counter-launched into a single dispersion compensated fiber with a length of 1 km, a high normal dispersion of −130 ps nm^−1^ km^−1^, and a low dispersion slope of −0.15 ps nm^−2^ km^−1^ for spectral broadening. The two combs are mixed and split into two beams for photoacoustic detection and power normalization, respectively.

### Influence of the mismatch between the comb line and cavity mode

The finesse of a Fabry–Pérot cavity consisting of two identical high-reflectivity mirrors is determined by $\pi \sqrt{R}/(1-R)$, where *R* is the reflectivity of the cavity mirrors. By measuring the reflectivity using the cavity-ring down method, the optical cavity used in this work has a finesse of 4078. Considering the cavity length of 18 cm, the *FSR* is determined to be ~833 MHz ($FSR=c/2L$, where *c* is the speed of light and *L* is the cavity length). The cavity mode can be described by a Lorentzian line-shape:

1 $$y=\frac{1}{\pi }\times \frac{\omega }{{(x-{x}_{c})}^{2}+{\omega }^{2}}$$

where *ω* and (*x−x*_*c*_) are the full width at half maximum (FWHM) and the frequency shift relative to the line-center of the cavity mode, respectively. For the optical cavity used in this work, the FWHM of the cavity mode is 204 kHz at the wavelength near 1531 nm. Selecting a center frequency difference of 5.5 kHz via AOMs and a repetition frequency difference of 30 Hz, the largest frequency among the dual-comb multiheterodyne beats is 8.6 kHz. Provided that one train of frequency combs is in perfect resonance with the cavity modes, the maximum frequency mismatch between the counterpart of the dual-comb and the cavity mode is 8.6 kHz. Hence, this corresponds to the intensity attenuation of only 0.66% according to Eq. (1).

### Evaluation of intracavity dual-comb power

The comparison of the single-pass and cavity-enhanced DCS signal in Fig. 2 proves a power enhancement of 924 times. The incident power of the dual-comb source is 30 mW and the total number of comb line pair is about 213. Hence, the average power is (30 mW × 924)/213 ≈ 130 mW for each pair of comb lines. The optical cavity (finesse 4078 in vacuum) theoretically leads to an intracavity power enhancement by a factor of 1299. Considering the finesse degradation induced by 10 ppm C_2_H_2_, the cavity enhancement factor is reduced to 1016. Hence, the comparison of the theoretical enhancement factor and the experimental one indicates a coupling efficiency of 91% for the dual-comb light.

### Signal-to-noise ratio evaluation

The strongest absorption line of the target species is selected for evaluating the SNR of the spectroscopic gas detection. In our cavity-enhanced photoacoustic DCS, the corresponding radio frequencies for C_2_H_2_, NH_3_, CO are located at 5290 Hz, 4480 Hz and 6430 Hz, respectively. The noise is measured in the same way by filling the gas cell with pure N_2_.

### Supplementary information

Supplemental
