# Supplementary material for: Cavity-enhanced photoacoustic dual-comb spectroscopy
Source: Light Sci Appl. 2024 Jan 5;13:11. doi: 10.1038/s41377-023-01353-6 (PMC10767139; doi:10.1038/s41377-023-01353-6)
Supplement: Supplementary file 1 — Supplemental [file 41377_2023_1353_MOESM1_ESM.docx]

Supplementary Information for

**Cavity-enhanced photoacoustic dual-comb spectroscopy**

Zhen Wang^1,^^†,*^, Qinxue Nie^1,†^_,_ Haojia Sun^1^, Qiang Wang^2,*^, Simone Borri^3^, Paolo De Natale^3^, and Wei Ren^1,*^

^1^Department of Mechanical and Automation Engineering, The Chinese University of Hong Kong, New Territories, Hong Kong SAR, China

^2^State Key Laboratory of Applied Optics, Changchun Institute of Optics, Fine Mechanics and Physics, Chinese Academy of Sciences, Changchun 130033, China

^3^CNR-INO – Istituto Nazionale di Ottica, and LENS – European Laboratory for Nonlinear Spectroscopy, 50019 Sesto Fiorentino, Italy

†These authors contributed equally.

*Email: wangzhen@link.cuhk.edu.hk; wangqiang@ciomp.ac.cn; renwei@mae.cuhk.edu.hk

**Supplementary Note 1: Locking dual-frequency combs with an optical cavity**

Fig. S1. Schematic of locking the dual-combs with an optical cavity. PBS: polarizing beam splitter; *f*_1_, *f*_2_: mode-matching lens; AOM: acousto-optic modulator; EOM: electro-optic modulator; NLL: narrow linewidth laser; PZT: piezoelectric transducer; PD: photodetector; FSR: free spectral range; PID: proportion-integration-differentiation; PDH: Pound-Drever-Hall locking.

The schematic of locking the dual-frequency combs with an optical cavity is illustrated in Fig. S1, which includes three PDH locking loops. The optical cavity of 180 mm in length includes two plane-concave mirrors with a radius of curvature of 150 mm. First, a portion of the seed laser used for dual-comb generation is shifted by an AOM. The shifted laser (*f*_c_+*f*_shift,1_), which has the same frequency as the central line of one comb, is transmitted through the EOM1 to generate a pair of sidebands (19 MHz). The laser polarization is made orthogonal to the dual-comb light to avoid optical crosstalk, and then combined via a polarization beam splitter (PBS) into the cavity. Two mode-matching lenses (*f*_1_: 50 mm and *f*_2_: 30 mm) are used to optimize the mode matching and maximize the coupling efficiency of the dual-comb light. The cavity reflection is picked up by PD2 and demodulated at 19 MHz to generate the PDH error signal, which is feedback to the seed laser via a proportion-integration-differentiation (PID) controller. By stabilizing *f*_c_, the central comb line is locked with the cavity.

It should be noted that the repetition rate of the electro-optic comb is precisely controlled by the RF signal generator and is quite stable. If the optical cavity is extremely stable, we are able to adjust the repetition rate of the frequency comb to match the FSR of the cavity. The cavity length can be stabilized by locking the optical cavity with a molecular absorption line. Here a CW narrow-linewidth fiber laser at 1531.58 nm is applied as an optical intermedium and is split into two branches. One branch is phase modulated (99 MHz) by EOM3 and passed through a reference gas cell filled with C_2_H_2_ at 10 Torr. The transmitted laser is detected by PD3 and demodulated by a PDH module. Based on the demodulated error signal, a PID controller stabilizes the laser frequency with the absorption line (P11) of C_2_H_2_. The other branch of the CW laser is modulated (13 MHz) by EOM2 and directed into the optical cavity via the PBS. The error signal derived from PD2 is used to lock the optical cavity to the CW laser by feeding back to the PZT that is attached to one cavity mirror. Note that the cavity reflection which includes the frequency-shifted seed laser and the CW laser is detected by PD2 simultaneously. Fortunately, the generated beatnotes are easily differentiated based on the different modulation frequencies used in each looping loop. Finally, the repetition rate of the frequency comb is adjusted to match the stabilized FSR so that all the comb lines are coupled into the cavity. All the signal generators are synchronized to a rubidium clock. According to our test, the comb-cavity locking can maintain beyond 12 hours with all the locking loops turned on.

**Supplementary Note 2: Characterization of acoustic resonators**


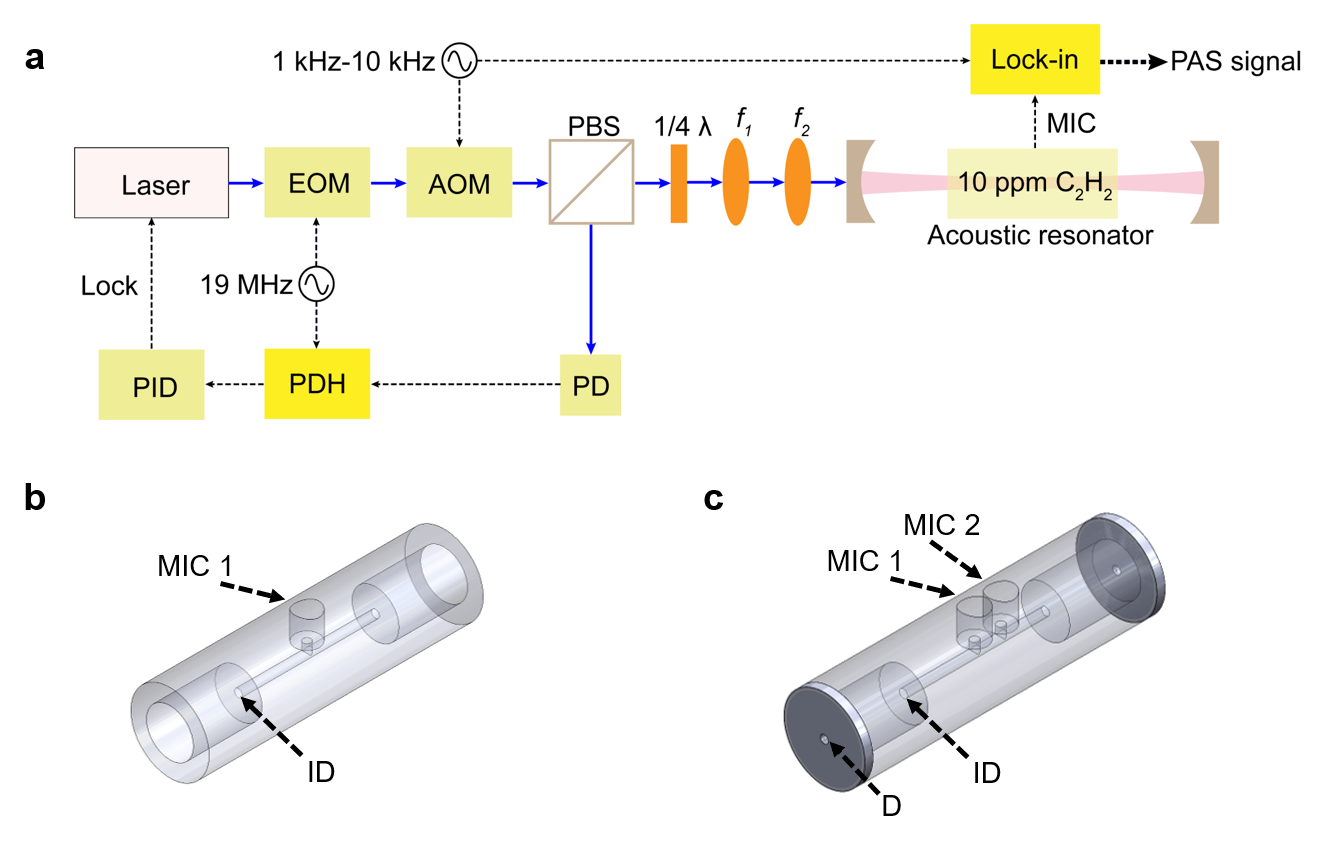


Fig. S2. **a** Experimental setup for characterizing the acoustic resonators. PBS: polarizing beam splitter; 1/4 λ: quarter-wave plate; *f*_1_, *f*_2_: mode-matching lens; AOM: acousto-optic modulator; EOM: electro-optic modulator; PD: photodetector; PID: proportion-integration-differentiation; PDH: Pound-Drever-Hall locking. **b** 3D drawing of the conventional acoustic resonator. MIC: microphone; ID: inner diameter. **c** 3D drawing of the flute-type acoustic resonator. D: diameter.

The frequency response is evaluated by measuring the cavity-enhanced photoacoustic signal of 10 ppm C_2_H_2_/N_2_ filled in the gas cell at different modulation frequencies of the laser intensity. The schematic of the setup is shown in Fig. S2a. A CW laser at 1531.58 nm with an emission power of ~6 mW is used to exploit the absorption line of C_2_H_2_. The laser is locked to the optical cavity (finesse: 4078) based on the PDH method (Supplementary Note 1). The laser intensity is modulated by an AOM to generate the photoacoustic signal, which is measured by a commercial microphone (EK-23133, Knowles). Fig. S2b and Fig. S2c demonstrate the 3D drawings of the traditional acoustic resonator and the flute-type resonator. The central longitudinal resonator has a diameter of 2 mm and a length of 35 mm; the buffering volumes have a length of 17.5 mm and ID of 12 mm. The acoustic resonator is installed between two cavity mirrors to enhance the amplitude of the acoustic wave. By sweeping the AOM frequency from 1 kHz to 10 kHz, the amplitude of the photoacoustic signal is demodulated by a lock-in amplifier (MFLI, Zurich Instruments). In this way, the frequency response can be obtained for different types of acoustic detectors.

**Supplementary Note 3: Normalization of** **photoacoustic DCS signal**

Fig. S3. **a** Comparison of photoacoustic DCS signals with and without normalization (top panel). The signal normalization takes into account the non-uniform distribution of three main factors over the spectral range including the comb intensity **b** the frequency response of the acoustic resonator **c** and the cavity finesse **d**.

The photoacoustic signals with and without normalization are shown in Fig. S3a, along with the absorption coefficient provided in the HITRAN database. The signal without normalization is derived from the original FFT results of the microphone signal. The signal normalization takes into account the non-uniform distribution of the main factors over the broad spectral range including the comb intensity, the cavity finesse and the frequency response of the acoustic resonator. Fig. S3b shows the reference multiheterodyne beatnotes of the dual-comb light, which is recorded by the photodetector during the photoacoustic measurement. Fig. S3c shows the measured frequency response of the acoustic resonator, which is fitted by a polynomial function. Fig. S3d shows the measured finesse of the optical cavity at the frequency of each comb line. A better agreement is observed between the normalized photoacoustic spectrum and the HITRAN database.

**Supplementary Note 4: Linear response and detection limit for NH_3_ and CO**


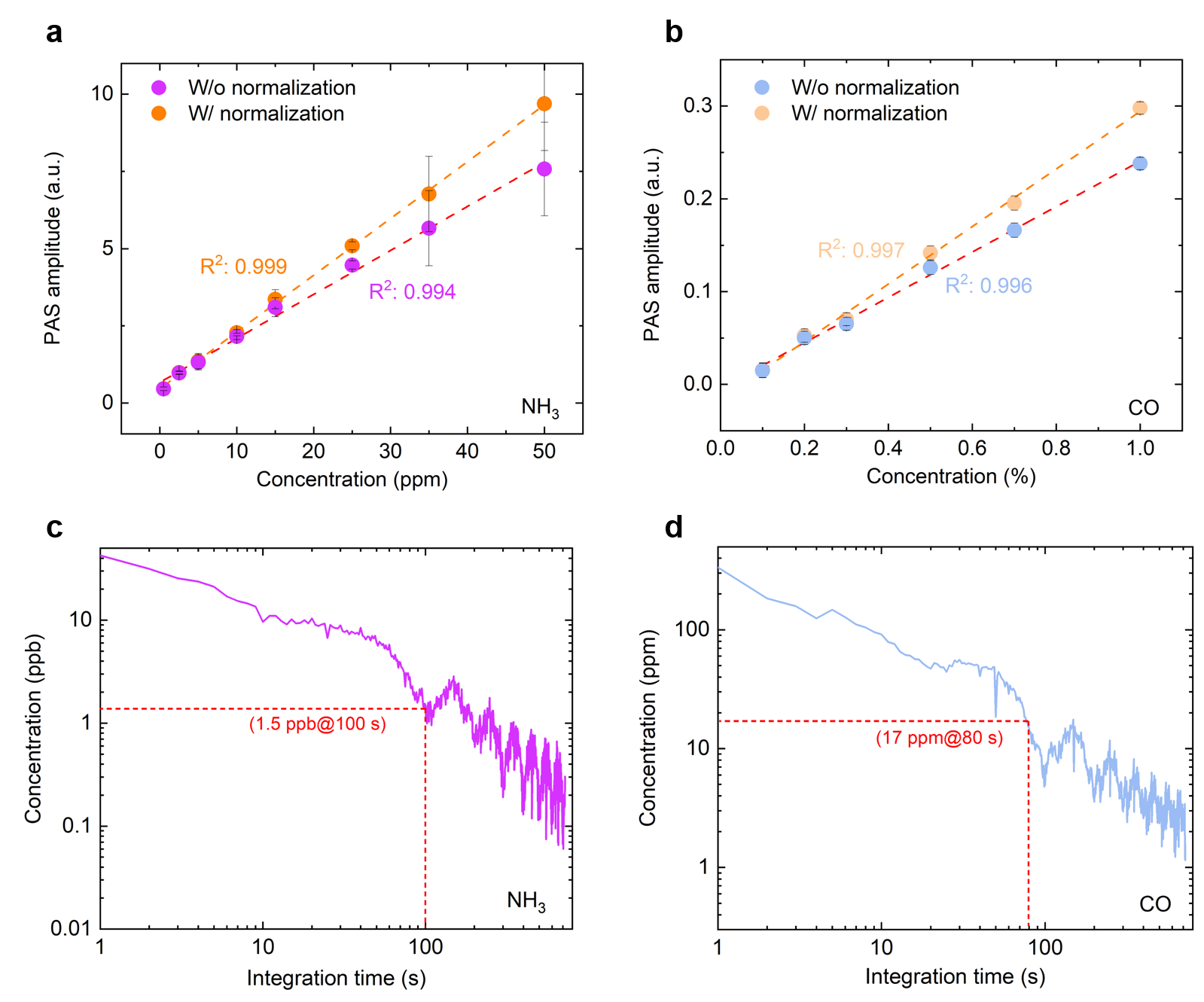


Fig. S4. Variation of the photoacoustic DCS signal with gas concentration: **a** NH_3_, and **b** CO. The vertical error bars (1-σ standard deviation) are calculated from the raw data, taken in a time interval of 60 sec. Allan deviation analysis is provided for **c** NH_3_, and **d** CO.

Photoacoustic DCS measurements of NH_3_ and CO were performed to evaluate the linear response of the system by selecting the blended lines (*^p^P*(5,3)*s*, *^p^P*(5,3)*a*) of NH_3_ at 195.731 THz with a line-strength of 1.35×10^-21^ cm^-1^ (molecules cm^-2^)^-1^ and the *R*(7) line of CO at 191.190 THz with a line-strength of 2.22×10^-23^ cm^-1^ (molecules cm^-2^)^-1^. The amplitudes of the signal at different gas concentrations are plotted in Fig. S4. The system shows a good linear response to NH_3_ (500 - 50000 ppb) and CO (1000 - 10000 ppm) with an R^2^ value > 0.99. Note that the larger error bar shown in Fig. S4a is mainly caused by the uncertainty of NH_3_ concentrations as NH_3_ is a kind of sticky molecule with apparent surface adsorption. The Allan-Werle deviation analysis is conducted by measuring the zero gas for 1 hour with all the locking loops turned on. The system demonstrates a noise equivalent concentration (NEC) of 1.5 ppb for NH_3_ at the averaging time of 100 sec and 17 ppm for CO at the averaging time of 80 sec, corresponding to 3.5×10^-10^ cm^-1^ and 4.9×10^-10^ cm^-1^ in noise equivalent absorption (NEA).
